# Time-Domain Simulation of Three Dimensional Quantum Wires

**DOI:** 10.1371/journal.pone.0153802

**Published:** 2016-04-28

**Authors:** Dennis M. Sullivan, Sean Mossman, Mark G. Kuzyk

**Affiliations:** 1 Department of Electrical and Computer Engineering, University of Idaho, Moscow, Idaho, United States of America; 2 Department of Physics and Astronomy, Washington State University, Pullman, Washington, United States of America; Tufts University, UNITED STATES

## Abstract

A method is presented to calculate the eigenenergies and eigenfunctions of quantum wires. This is a true three-dimensional method based on a direct implementation of the time-dependent Schrödinger equation. It makes no approximations to the Schrödinger equation other than the finite-difference approximation of the space and time derivatives. The accuracy of our method is tested by comparing it to analytical results in a cylindrical wire.

## Introduction

Quantum wires have become extremely important structures in the field of quantum nanodevices. They have found applications in such diverse fields as quantum electronics [1–5] and nonlinear optics [6–10]. This paper describes the use of the finite-difference time-domain (FDTD) method to determine the eigenenergies and eigenfunctions of quantum wires from the potential function [11–15]. Specifically, a method is described to accurately model long, thin wires without using excessive computer resources. Furthermore, a Bessel function method is presented that can be used to verify the calculations of the FDTD method.

An important application of this method is to nonlinear optics, where nonlinear susceptibilities are calculated from the quantum properties of the material. For example, transition moments, which are calculated from wave functions, and energies are used in a sum-over-states expression to get nonlinear susceptibilities. This approach was used to find optimized potentials [16, 17] as well as to analyze the character of a potential that gives the largest nonlinearity by analyzing ones made of a finite number for straight line segments, as demonstrated by Burke [18–20].

FDTD can be used to quickly calculate nonlinear susceptibilities, which can be used to optimize the nonlinear response by varying the shapes of arbitrary structures. In this work, we consider structures made of fused conducting cylinders because these are the types of structures that can be made using common fabrication techniques found in nanotechnology labs. As such, this work can be seen as providing the computational building blocks with applications to nonlinear optics, electronics, and related fields.

In the next section of this paper, we describe the implementation of the FDTD method and its use to calculate eigenenergies and eigenfunctions. This includes a description of a method to increase the resolution of the FDTD simulation by averaging the potential in cells bordering two different materials. We then present the results of determining the eigenenergies of a quantum wire at various radii along with the Bessel function results to confirm the accuracy. The flexibility of the method is demonstrated by modeling potentials that do not otherwise easily lend themselves to analysis. We end by summarizing the results of the paper and discussing future directions. S1 Appendix describes the Bessel function method.

## The Finite-Difference Time-Domain Method

We begin describing the FDTD implementation of the time-dependent Schrödinger equation [13, 14], which is written in the following form

$$\begin{array}{l} \frac{\partial \psi \left(x,y,z,t\right)}{\partial t}=i\frac{\hbar }{2m_{e}}\left[\frac{\partial^{2}\psi \left(x,y,z,t\right)}{\partial x^{2}}+\frac{\partial^{2}\psi \left(x,y,z,t\right)}{\partial y^{2}}+\frac{\partial^{2}\psi \left(x,y,z,t\right)}{\partial z^{2}}\right]\\ \;-\frac{i}{\hbar }V\left(x,y,z\right)\psi \left(x,y,z,t\right). \end{array}$$ (1)

We separate *ψ*(*x*,*y*,*z*,*t*) into real and imaginary components:

$$\psi \left(x,y,z,t\right)=\psi_{real}\left(x,y,z,t\right)+i\cdot \psi_{imag}\left(x,y,z,t\right).$$ (2)

Inserting Eq 2 into Eq 1 leads to two coupled equations:

$$\frac{\partial \psi_{real}\left(x,y,z,t\right)}{\partial t}=-\frac{\hbar }{2m_{e}}\nabla^{2}\psi_{imag}\left(x,y,z,t\right)+\frac{1}{\hbar }V\left(x,y,z\right)\psi_{imag}\left(x,y,z,t\right),$$ (3 a)

$$\frac{\partial \psi_{imag}\left(x,y,z,t\right)}{\partial t}=\frac{\hbar }{2m_{e}}\nabla^{2}\psi_{real}\left(x,y,z,t\right)-\frac{1}{\hbar }V\left(x,y,z\right)\psi_{real}\left(x,y,z,t\right).$$ (3 b)

To code these equations, we take the finite-difference approximations in space and time which results in the following two coupled equations:

$$\begin{array}{l} \psi_{real}^{k+1}\left(m,n,l\right)=\psi_{real}^{k}\left(m,n,l\right)+\frac{\Delta t}{\hbar }V\left(m,n,l\right)\psi_{imag}^{k+1/2}\left(m,n,l\right)\\ \;-\frac{\hbar }{2m_{e}}\frac{\Delta t}{{\left(\Delta x\right)}^{2}}\left[\begin{array}{l} \;\psi_{imag}^{k+1/2}\left(m+1,n,l\right)+\psi_{imag}^{k+1/2}\left(m-1,n,l\right)\\ +\psi_{imag}^{k+1/2}\left(m,n+1,l\right)+\psi_{imag}^{k+1/2}\left(m,n-1,l\right)\\ +\psi_{imag}^{k+1/2}\left(m,n,l+1\right)+\psi_{imag}^{k+1/2}\left(m,n,l-1\right)-6\psi_{imag}^{k+1/2}\left(m,n,l\right) \end{array}\right], \end{array}$$ (4 a)

$$\begin{array}{l} \psi_{imag}^{k+3/2}\left(m,n,l\right)=\psi_{imag}^{k+1/2}\left(m,n,l\right)-\frac{\Delta t}{\hbar }V\left(m,n,l\right)\psi_{real}^{k+1}\left(m,n,l\right)\\ \;+\frac{\hbar }{2m_{e}}\frac{\Delta t}{{\left(\Delta x\right)}^{2}}\left[\begin{array}{l} \;\psi_{real}^{k+1}\left(m+1,n,l\right)+\psi_{real}^{k+1}\left(m-1,n,l\right)\\ +\psi_{real}^{k+1}\left(m,n+1,l\right)+\psi_{real}^{k+1}\left(m,n-1,l\right)\\ +\psi_{real}^{k+1}\left(m,n,l+1\right)+\psi_{real}^{k+1}\left(m,n,l+1\right)-6\psi_{real}^{k+1}\left(m,n,l\right) \end{array}\right]. \end{array}$$ (4 b)

In Eq 4 integer indices *m*,*n*.*l* representing the positions in a matrix have replaced the Cartesian coordinates *x*,*y*,*z*, respectively, in Eq 3. Similarly, the time step *k* has replaced *t*. Once the cell size Δ*x* is chosen, the time step Δ*t* must also be chosen so the constants preceding the spatial Laplacian are small enough to maintain stability [21]. The alternate iteration of the real and imaginary Eq (4a) and (4b) simulates the behavior of the waveform propagating in time. Details are available in the literature [11–15]. It is also necessary to be able to absorb outgoing waveforms to prevent them from being reflected back into the problem space and interfering with the simulation. This is accomplished with a perfectly matched layer (PML) [22]. The PML effectively adds artificial impedance between the real and imaginary parts of the waveform. The PML being used is based on Zheng [23] as described in a previous paper [24], so details will not be repeated here.

**Fig 1:**
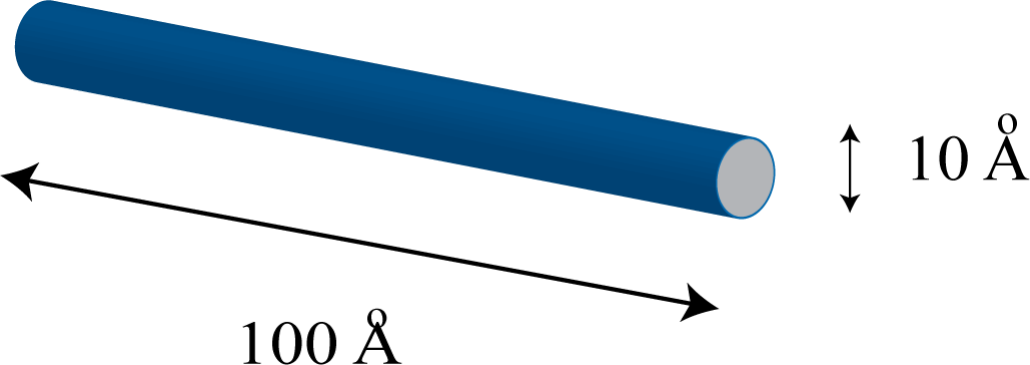
A quantum wire that is 100 Angstroms long and 10 Angstroms or less in diameter.

**Fig 2:**
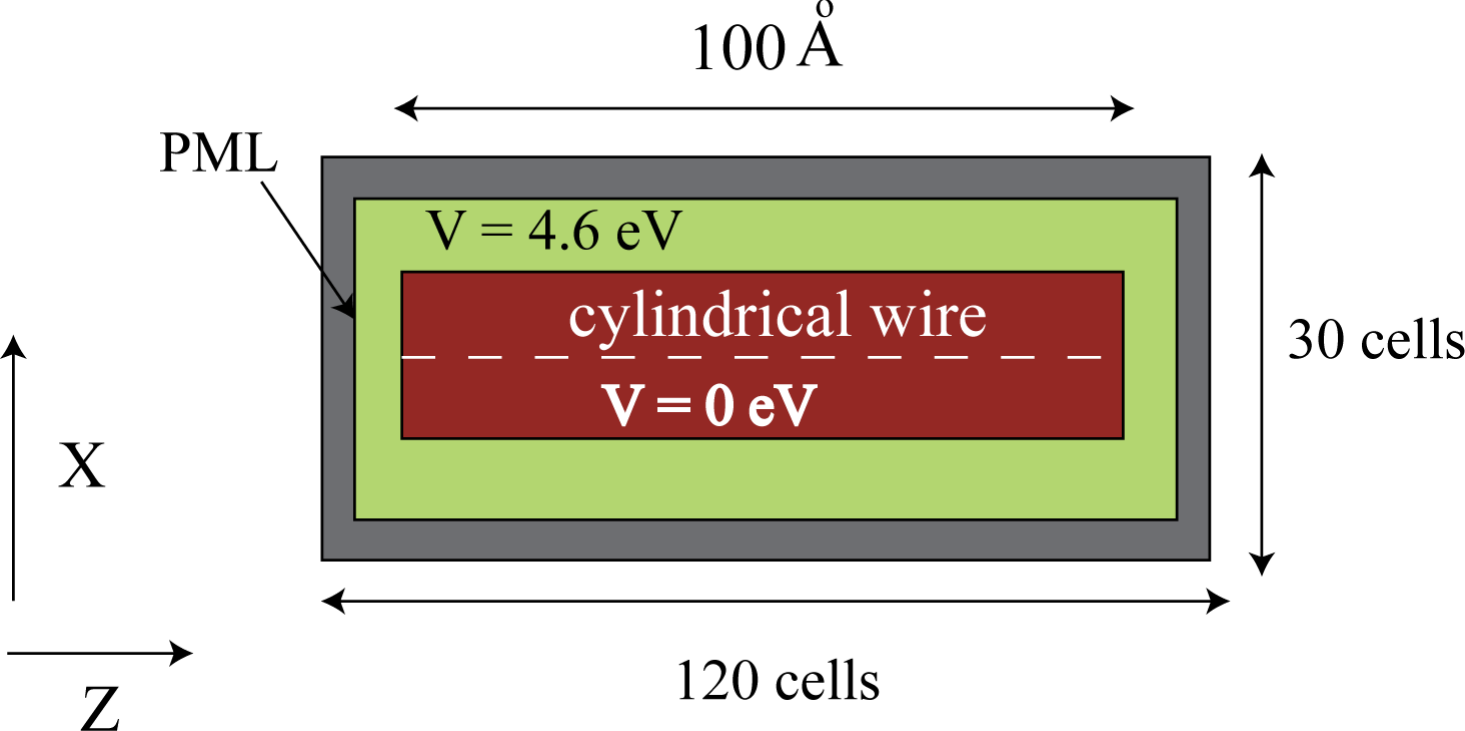
The problem space for the simulation of a cylindrical quantum wire. The cells are one Angstrom cubed. The entire three-dimensional space is 30x30x120 cells. (The Y direction is not shown.)

**Fig 3:**
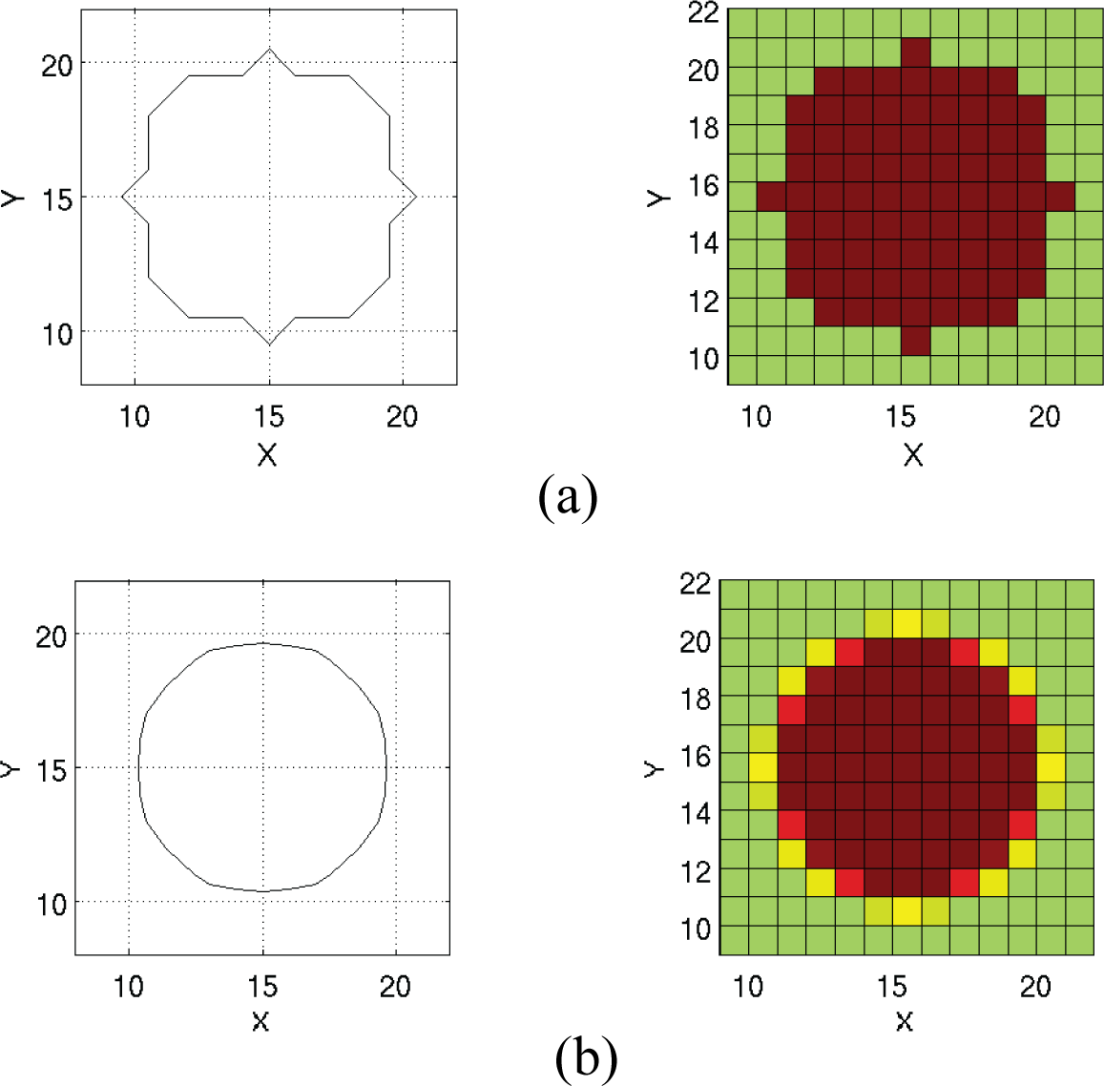
Illustration of the difference between the two methods of specifying the potential. Green represents a potential of 4.6 eV while red represents zero potential. The shades of orange represent weighted averages between the two. (a) The “in or out” method; (b) Averaging method.

Fig 1 illustrates the quantum wires to be simulated. The aspect ratio is at least ten to one, so we will use a length of 100 Angstroms, and a diameter of 10 Angstroms or less. We assume that the inside of the wire is at zero potential and the background medium is 4.6 eV corresponding to the work function of silver. Fig 2 illustrates the problem space being used to simulate quantum wires. Normally, the potential of a quantum structure is specified by setting the *V* parameter in Eq 4a and 4b) to the potential corresponding to the location in the structure being simulated. As an example, the cross-section of a quantum wire of radius 5 Angstroms using cells of one Angstrom might appear as shown in Fig 3A. The red indicates the inside of the wire which is at zero potential, while the green indicates the surrounding potential at *V* = 4.6 eV. Grey represents the PML. Borrowing from a method developed for electromagnetic simulation [25], a more accurate representation of the boundaries can be achieved by averaging the fraction of each material that is in a cell and assigning the value of potential accordingly. The results are shown in Fig 3B.

## Determining the Eigenenergies and Eigenfunctions

The FDTD method can be used to determine the eigenenergies and eigenstates of a potential that does not otherwise lend itself to an analytic solution. Any quantum wave function in a given quantum system can be written in the following manner,

$$\psi \left(r,t\right)=\sum_{n=0}^{N}\phi_{n}\left(r\right)e^{-i\left(\varepsilon_{n}/\hbar \right)t},$$ (5)

where the *ϕ*_*n*_(*r*) are the eigenfunctions of the system and the *ε*_*n*_ are the corresponding eigenenergies [11]. We can write the state variable in this fashion even if we do not know the eigenfunctions and eigenenergies.

The eigenenergies can be determined by monitoring the time-domain data at one point in the problems space, say *r*_0_, and then taking the Fourier transform,

$$F\left\{\psi \left(r_{0},t\right)\right\}=\int_{-\infty }^{\infty }dt\left[\sum_{n=0}^{N}\phi_{n}\left(r_{0}\right)e^{-i\left(\varepsilon_{n}/\hbar \right)t}\right]e^{i\omega t}=\sum_{n=0}^{N}\phi_{n}\left(r_{0}\right)\delta \left(\omega -\frac{\varepsilon_{n}}{\hbar }\right).$$ (6)

The last step results from the following Equations:

$$\int_{-\infty }^{\infty }e^{-i\left(\omega_{n}-\omega_{m}\right)t}dt=\{\begin{array}{c} 1\;n=m\\ 0\;n\neq m \end{array}.$$ (7)

The above transform produces a series of delta functions in the frequency domain corresponding to the eigenenergies.

**Fig 4:**
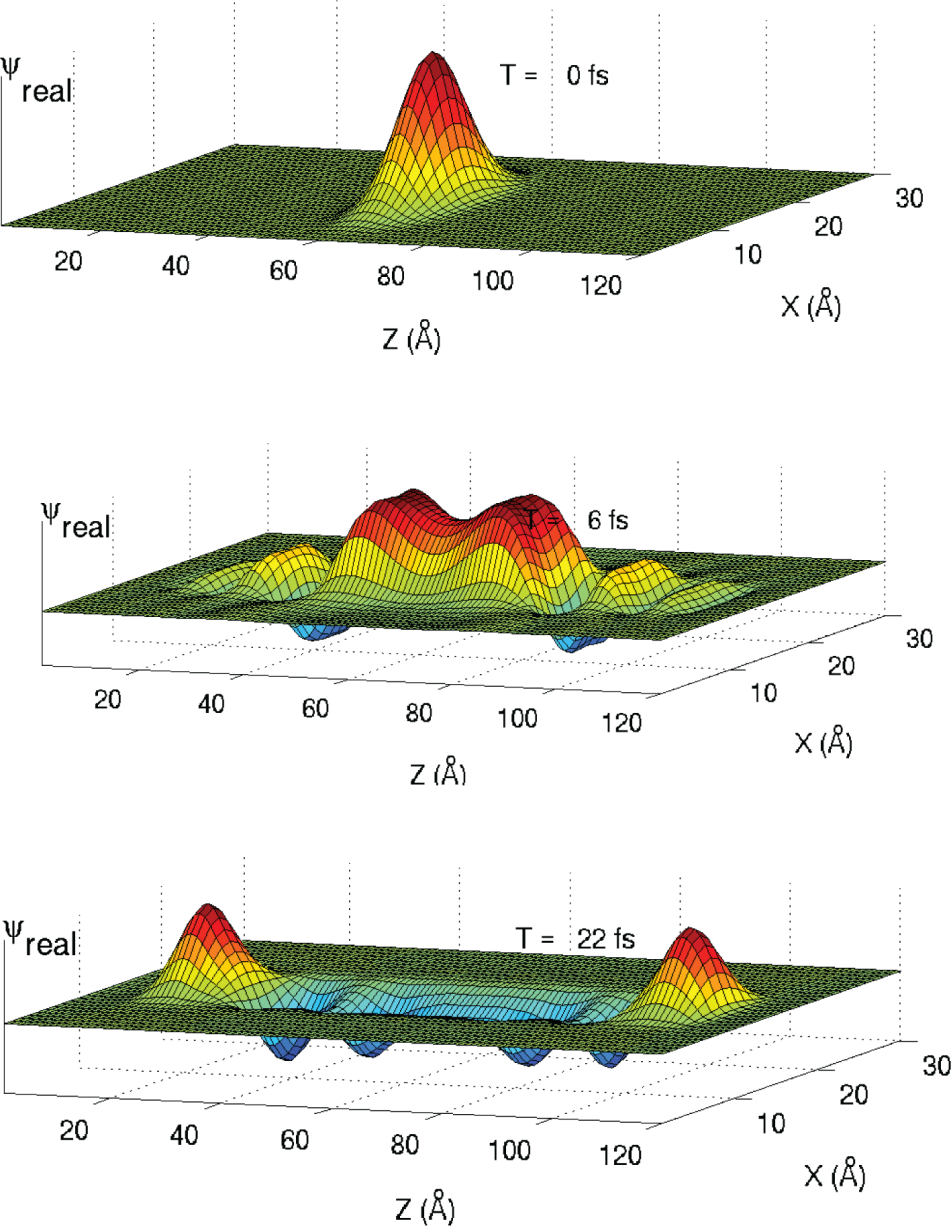
A test function is initialized in the problems space. As the FDTD simulation proceeds, the waveform spreads out.

The simulation starts by initializing a test function in the potential, as shown in Fig 4. The test function is a narrow Gaussian pulse. It chosen to ensure that it contains all the eigenfunctions of the structure being analyses. As the time-domain simulation proceeds, the pulse spreads out, some of it being absorbed by the PML. However, most of the waveform remains in the wire. During the simulation, the time-domain data at the original source point is saved. All the waveform simulations in this paper are in three dimensions. Since only two dimensions are displayed, we show the middle of the problems space in the *y* direction.

**Fig 5:**
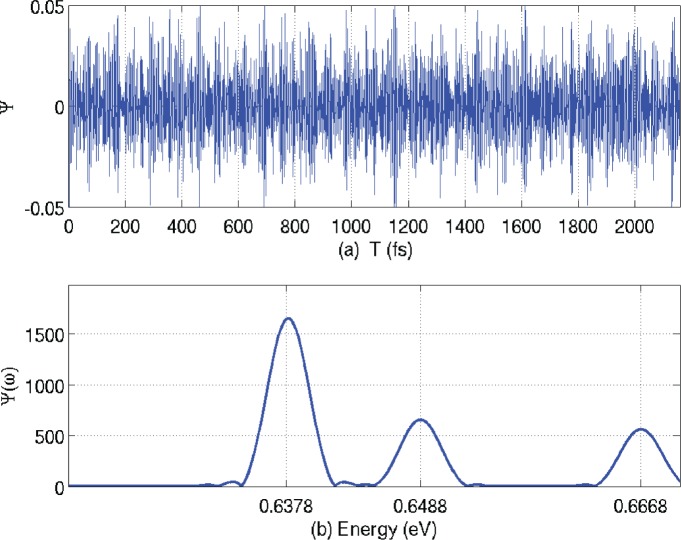
**(a) The stored time-domain data; (b) the Fourier transform.** Frequency has been converted to energy.

After 100,000 iterations, the program is halted. The stored time-domain data is shown in Fig 5(A), and the Fourier transform of this data is shown in Fig 5(B). The first two eigenenergies appear at 0.6378 and 0.6488.

**Fig 6:**
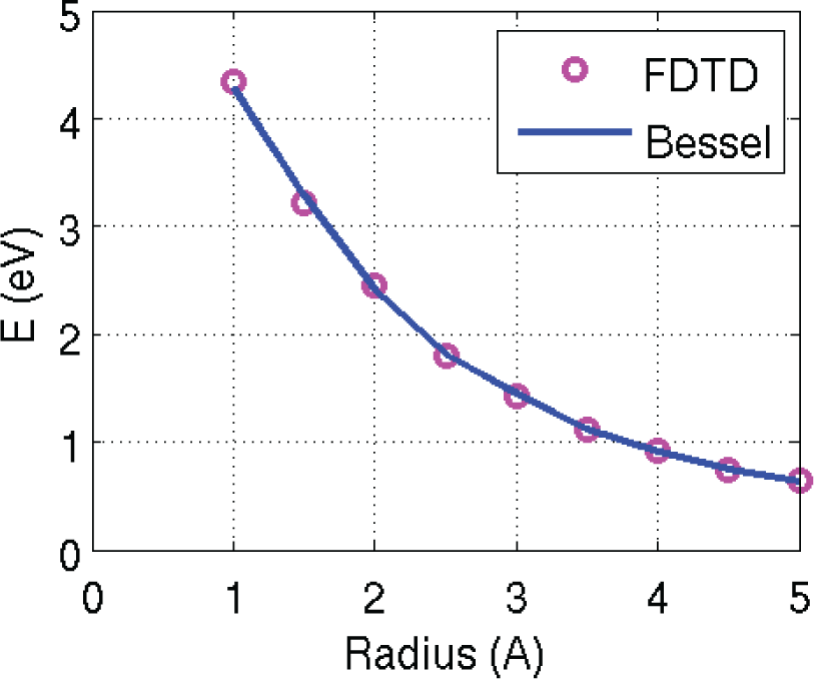
Comparison of the FDTD method vs. Bessel function in calculating ground state energies of the 100 Angstrom cylinder as a function of the radius of the cylinder.

In order to verify the accuracy of the FDTD technique, an analytic method based on Bessel functions has been developed and is described in S1 Appendix of this paper. Table 1 shows a comparison of the FDTD determination of the ground state energies of the quantum wire of Fig 1 for various radii versus the Bessel function method. The results are also graphed in Fig 6.

**Table 1:** Ground state energies for the cylindrical wire illustrated in Fig 1 for various radii as determined by the FDTD method and the Bessel function method.

Radius (A)	FDTD (eV)	Bessel (eV)	Difference (%)
5.0	0.6378	0.6297	1.3
4.5	0.7504	0.7489	0.8
4.0	0.9207	0.9051	1.7
3.5	1.1135	1.1148	-1.2
3.0	1.4315	1.4041	1.9
2.5	1.7983	1.8151	-0.9
2.0	2.4590	2.4131	1.9
1.5	3.2131	3.2734	-1.8
1.0	4.3439	4.2797	1.5

To determine the eigenfunction *ϕ*_*m*_(*r*) corresponding to an eigenenergy *ε*_*m*_, we take the discrete Fourier transform of the state variable at the frequency *ω*_*m*_ = *ε*_*m*_ / ℏ at every point in the problems space:

$$\begin{array}{l} DFT{\left\{\psi \left(r,t\right)\right\}}_{\omega =\varepsilon_{m}/\hbar }=\int_{-\infty }^{\infty }dt\left[\sum_{n=0}^{N}\phi_{n}\left(r\right)e^{-i\left(\varepsilon_{n}/\hbar \right)t}\right]e^{i\left(\varepsilon_{m}/\hbar \right)t}\\ =\int_{-\infty }^{\infty }dt\left[\sum_{n=0}^{N}\phi_{n}\left(r\right)e^{-i\left(\left(\varepsilon_{n}-\varepsilon_{m}\right)/\hbar \right)t}\right]=\phi_{m}\left(r\right). \end{array}$$ (8)

**Fig 7:**
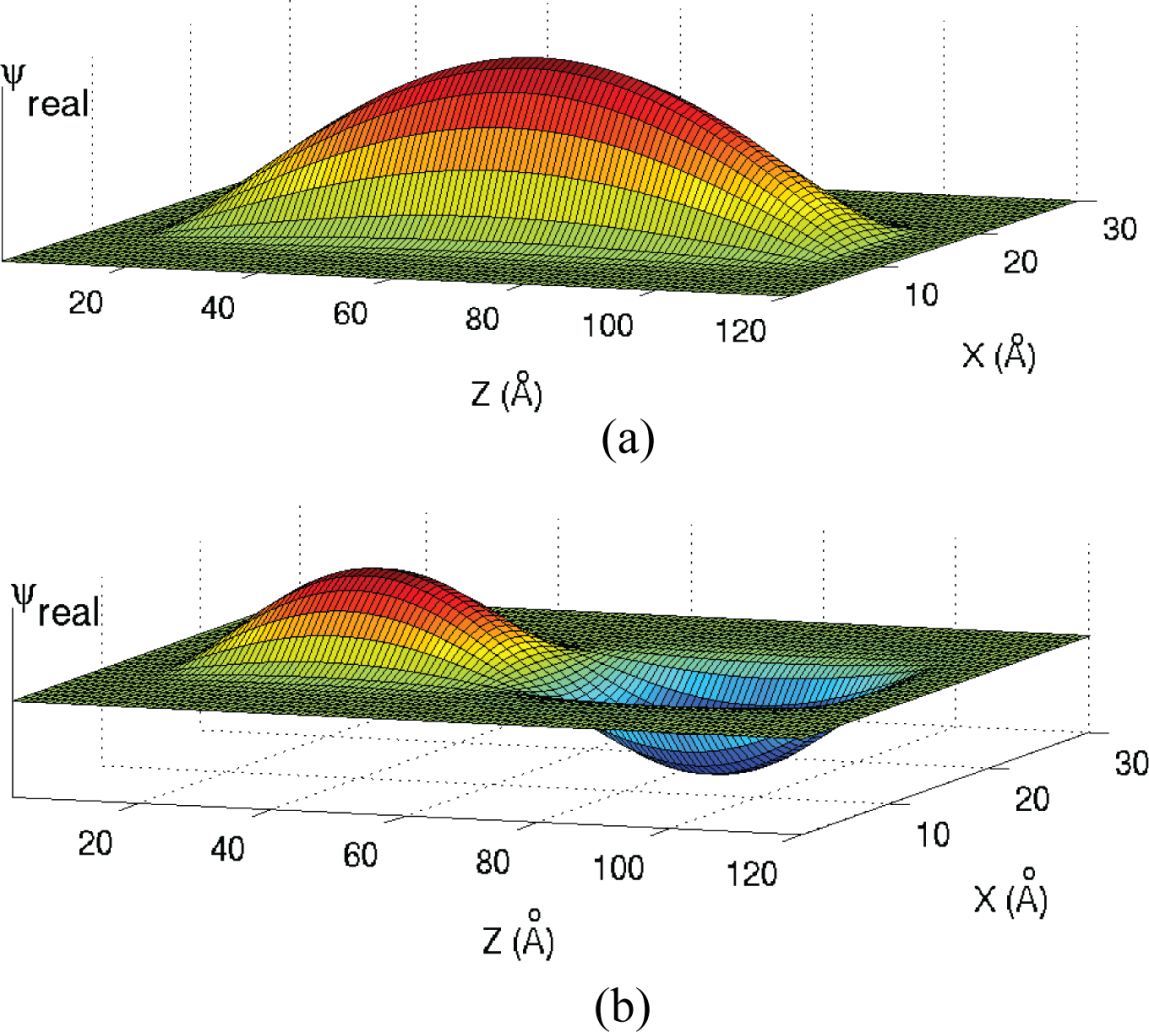
The first two eigenfunctions corresponding to the first two eigenenergies for the cylinder of Fig 1 with a radius of 5 Angstroms. (a) 1^st^ eigenstate at 0.6378 eV; 2^nd^ eigenstate at 0.6488 eV.

To construct the ground state eigenfunction at *ε*_0_ = 0.6378 *eV* the original test function is initialized in the problem space as in Fig 4. However, as the simulation proceeds, the discrete Fourier transform at the frequency corresponding to 0.6378 eV is taken at every cell in the problem space. The process is repeated for the second eigenstate at 0.6488 eV. The results are shown in Fig 7.

## Other Wire Configurations

In the previous section the simulation examples used simple cylinders to establish the accuracy by comparison with an analytic solution. The true strength of the FDTD method is that it can be used on a potential that is not necessarily a simple, regular structure. Two examples are given in this section.

**Fig 8:**
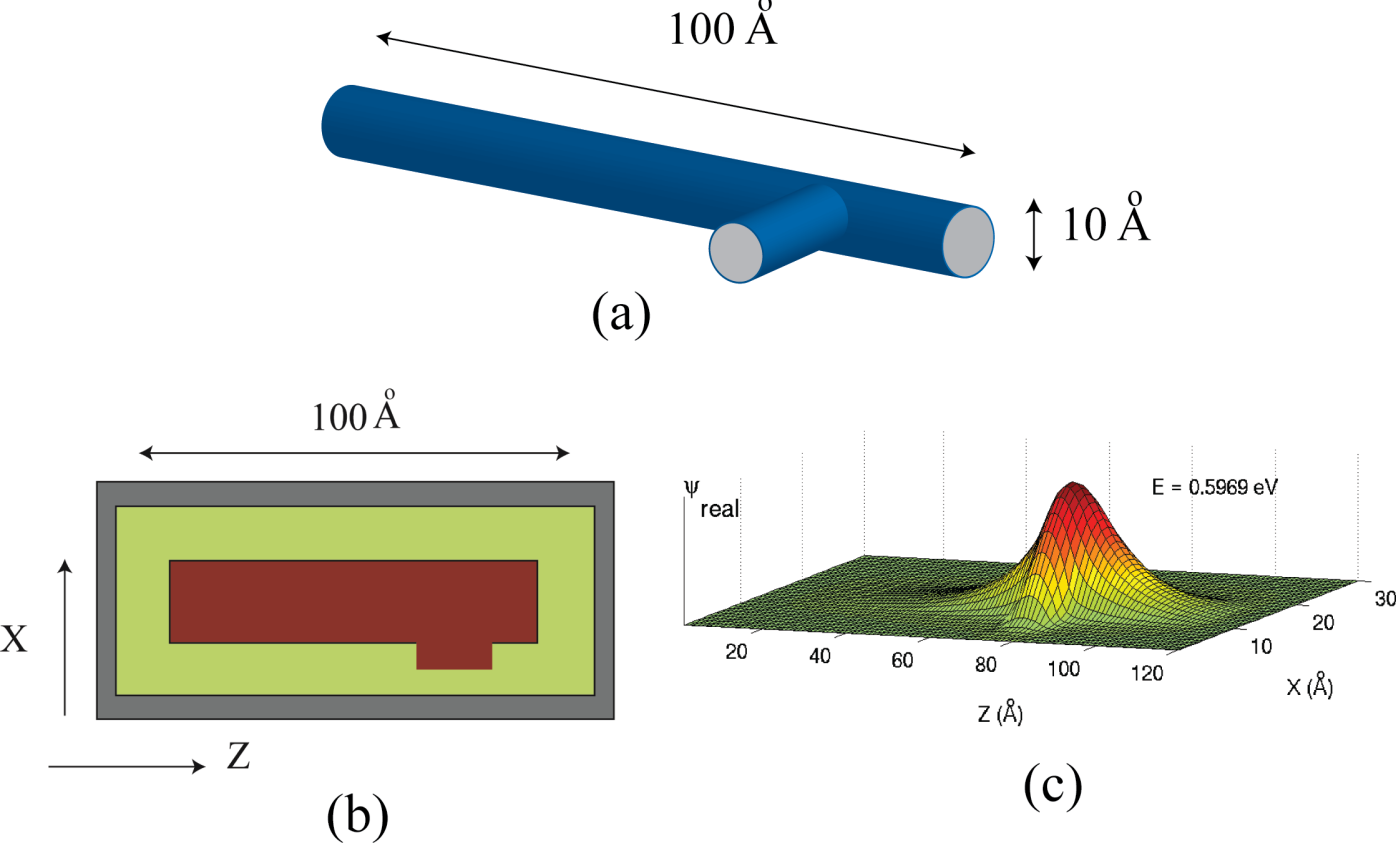
**A 100 Angstrom quantum wire with a small perpendicular wire attached;** (a) Diagram of the wire; (b) Diagram of the problem space; (c) The ground state eigenfunction.

By adding short cylinders perpendicular to the main cylinder, it may be possible to substantially increase the hyperpolarization of the system [6]. One possible configuration is illustrated in Fig 8(A), where a small cylinder seven Angstroms long was added to the main cylinder. Fig 8(B) shows the corresponding problem space. Fig 8C illustrates the corresponding ground state eigenfunction.

**Fig 9:**
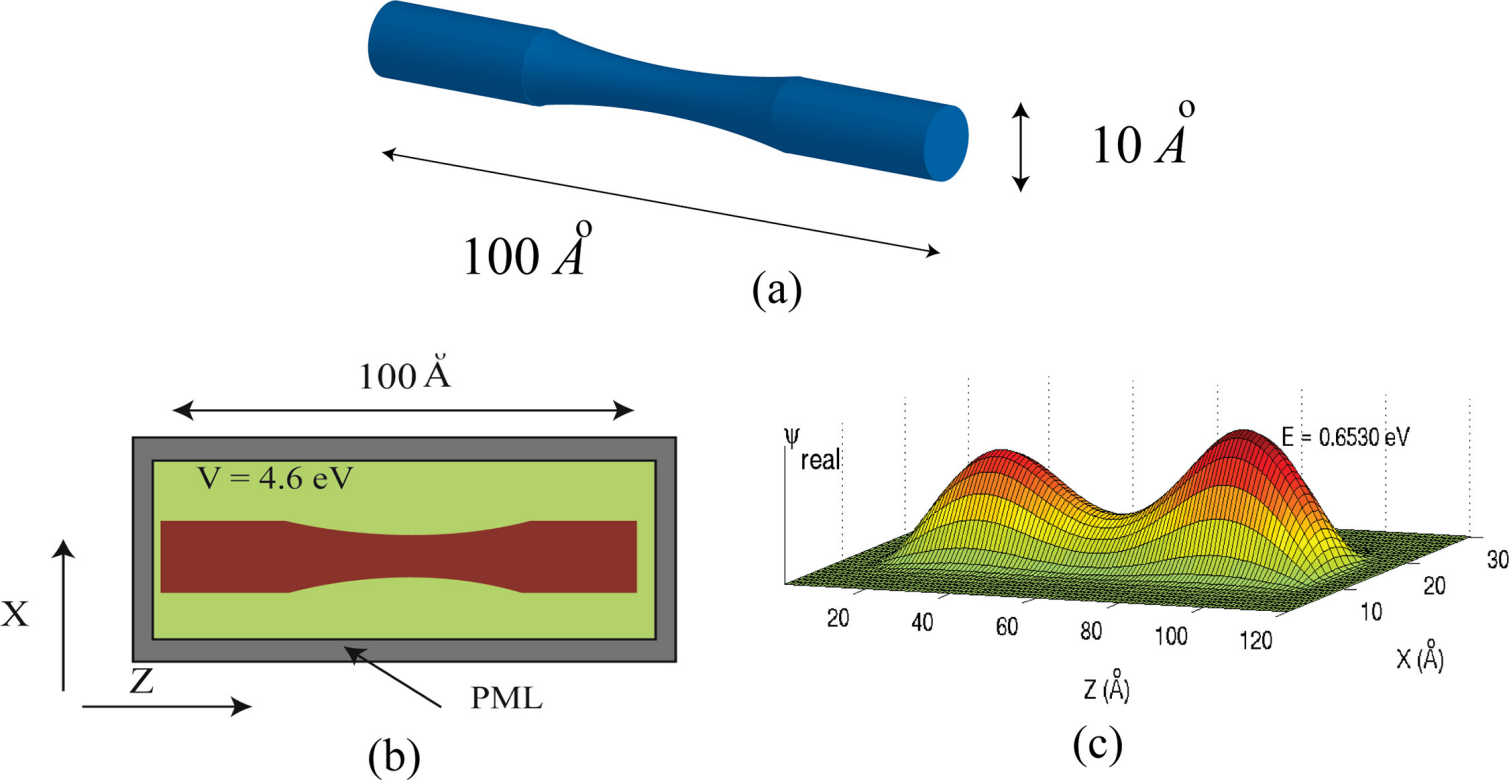
**(a) A tapered 100 Angstrom cylindrical wire.** (a) Diagram of the tapered wire; (b) Diagram of the problem space; (c) The ground state eigenfunction. Fig 8C shows that there is some probability that a particle exists in the small perpendicular appendage of the wire illustrated in Fig 8A. The eigenfunction in Fig 9C illustrates that a particle at the ground state energy is equally likely to be found at each end of the wire of Fig 9A, but very unlikely to be found in the middle.

Another example is shown in Fig 9(A) where the cylinder is tapered slightly in the middle. The tapering has been proposed as a method of controlling transport in nanowire MOSFETs (metal oxide semiconductor, field effect transistors.) [2].

## Conclusion

We have shown that the FDTD method can be used to determine the eigenenergies and eigenfunctions of quantum wires on size scales that are of interest to nanotechnologists. We have applied the FDTD method to solve the simple cylinder, which has analytic solutions in the form of Bessel functions. The two methods give eigenenergies that are accurate to better than two percent. Comparisons with other methods were not made. The more complex structures that we have calculated here cannot be tested against analytic solutions. However, given that they are made of merged cylinders, we expect that the accuracy will be comparable.

The simulations described in this paper were done on an HP DL140 with eight cores, a high end workstation, and does not represent extraordinary computational resources. A typical simulation to determine the eigenenergy and eigenfunction requires about 40 seconds. Note that these simulations require no assumptions about the shape of the object, so computational speeds are not affected by the shape.

Future projects include simulating multiple particles in a quantum wire. The FDTD method has already been shown to be capable of simulating two particles in a quantum dot [26].

## Supporting Information

S1 Appendix Bessel Function Determination of Eigenenergies of a Cylindrical Wire. (DOC)
