# Supplementary material for: Time-Domain Simulation of Three Dimensional Quantum Wires
Source: PLoS One. 2016 Apr 28;11(4):e0153802. doi: 10.1371/journal.pone.0153802 (PMC4849664; doi:10.1371/journal.pone.0153802)
Supplement: S1 Appendix — (DOC) [file pone.0153802.s001.doc]

**Appendix A—Bessel Function Determination of Eigenenergies of a Cylindrical Wire**

The time-independent Schrödinger equation is

. (9)

We start by rewriting it as follows,

$\nabla^{2}\psi+k^{2}\psi=0$, (10)

where

. (11)

In cylindrical coordinates, Eq. (10) can be written as [1]

$\frac{1}{R\left(\rho\right)\rho}\frac{\partial}{d\partial}\left(\rho\frac{\partial}{d\partial}R\left(\rho\right)\right)-n^{2}-k_{z}^{2}+k^{2}=0$,

where *n* is the quantum number from the dependence, which is the angle in the transverse direction in cylindrical coordinates. We will only be interested in the ground state solution in the $\rho$ direction, so we take *n=0* and $k_{z}$ as the quantum number from the z direction dependence. Since we are left with only $\rho$ and *z* parameters, we write

$k^{2}=k_{z}^{2}+k_{\rho}^{2}$,

or

$k_{\rho}^{2}=k^{2}-k_{z}^{2}$.

This yields

$\frac{1}{R\left(\rho\right)\rho}\frac{\partial}{d\partial}\left(\rho\frac{\partial}{d\partial}R\left(\rho\right)\right)+k_{\rho}^{2}=0$. (12)

The solutions to Eq. (12) are the Bessel functions, when

. (13)

The value of$E_{z}$ is determined by the eigenenergies of the one-dimensional well in the z direction. These can be determined by a program that does an eigenfunction decomposition of a matrix representing the Hamiltonian [2]. For the case shown in Fig. 1, the first two Z-direction eigenergies are $E_{z0}=0.00363 eV$ and $E_{z1}=0.01450 eV$.

Inside the cylinder, where *V = 0,*

, (14)

and when $E>E_{z}$, $k_{\rho}$ is real. The solutions are the Bessel functions of the first and second kind, $J_{n}\left(k_{\rho}\rho\right)$ and $N_{n}\left(k_{\rho}\rho\right)$, respectively. $N_{n}\left(k_{\rho}\rho\right)$ diverges at $k_{\rho}\rho=0$, so it is eliminated. Since *n =* 0 for the ground state we are left with $J_{0}\left(k_{\rho}\rho\right)$ as the solution.

Outside the cylinder, where $V=V_{1}$ and $E-V_{1}<0$, Eq. (12) shows that $k_{\rho}$ is imaginary. The solution is the modified Bessel function of the second kind, $K_{0}\left(k_{\rho}\rho\right)$, which decays exponentially, so solutions are of the form

. (15)

The continuity conditions at the edge of the cylinder,$\rho=a$, dictate that,

$EJ_{0}\left(k_{0}a\right)=FK_{0}\left(k_{1}a\right)$, (16a)

and

$EJ_{0}'\left(k_{0}a\right)=FK_{0}'\left(k_{1}a\right)$. (16b)

The derivatives are most easily calculated using [3],

$J_{0}'\left(x\right)=-J_{1}\left(x\right)$, (17a)

$K_{0}'\left(x\right)=-K_{1}\left(x\right)$. (17b)

Therefore,

$J_{0}'\left(k_{0}a\right)=\left.\frac{dJ_{0}\left(k_{0}\rho\right)}{d\rho}\right|_{\rho=a}=-k_{0}J_{1}\left(k_{0}a\right)$, (18a)

and

$K_{0}'\left(k_{1}a\right)=\left.\frac{dK_{0}\left(k_{1}\rho\right)}{d\rho}\right|_{\rho=a}=-ik_{1}K_{1}\left(k_{1}a\right)$. (18b)

The continuity equations then become

$EJ_{0}\left(k_{0}a\right)=FK_{0}\left(k_{1}a\right)$, (19a)

and

$Ek_{0}J_{1}\left(k_{0}a\right)=-Fik_{1}K_{1}\left(k_{1}a\right)$. (19b)

Following a method suggested by Griffiths [4], we divide Eq. (19b) by Eq. (19a) to obtain,

$\frac{J_{0}\left(k_{0}a\right)}{k_{0}J_{1}\left(k_{0}a\right)}=\frac{K_{0}\left(k_{1}a\right)}{-ik_{1}K_{1}\left(k_{1}a\right)}$.

Defining

$G(E)=\frac{k_{0}J_{1}\left(k_{0}a\right)}{-ik_{1}J_{0}\left(k_{0}a\right)}\frac{K_{0}\left(k_{1}a\right)}{K_{1}\left(k_{1}a\right)}=1$, (20)

for a given $k_{z}$, we use the definitions of $k_{0}$ and $k_{1}$ to determine the values of *E* that result in *G(E) = 1.* The results are shown Figure A for the first two eigenstates of a 100 Angstrom long cylinder with a radius of 5 Angstroms.

**Figure A.** Solution of Eq. (20) for the two $E_{z}$ values that determine the first two eigenenergies of the cylinder.


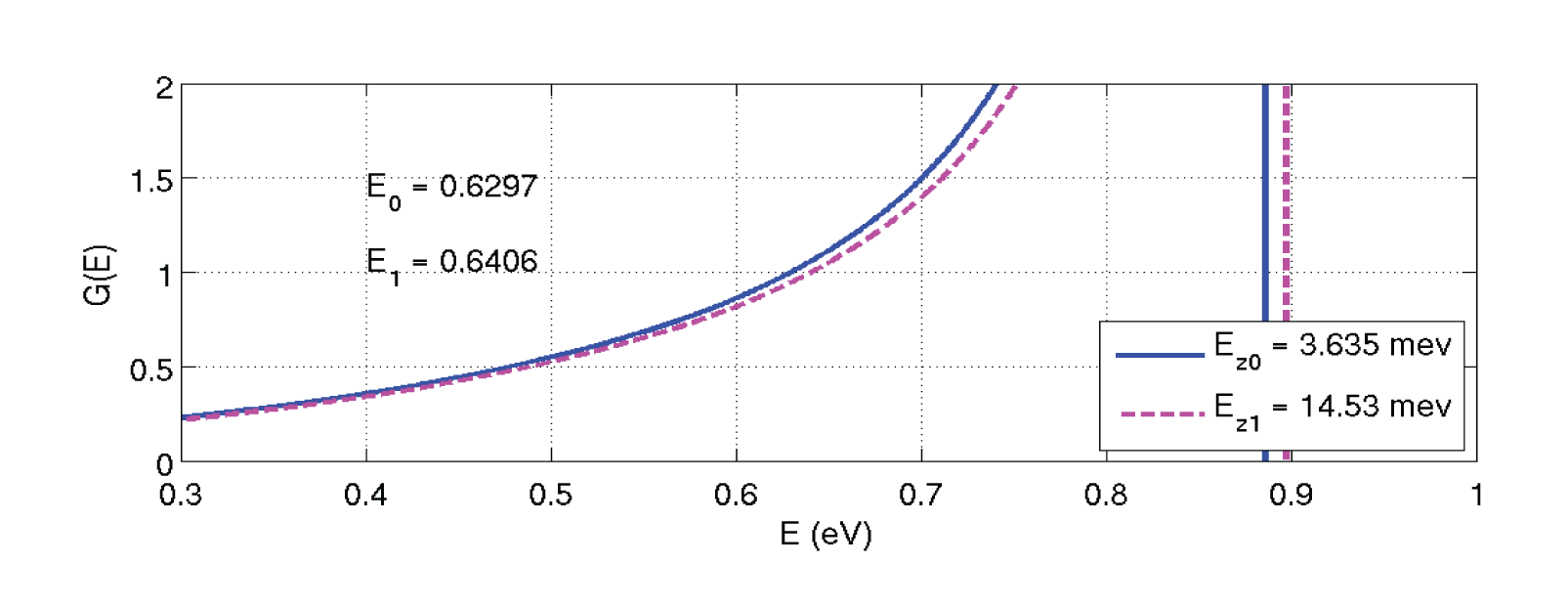


**References:**

1. Harrington RF. Time-harmonic electromagnetic fields*,* McGraw-Hill; 1961.

2. Datta S. Quantum Transport---Atom to Transistor. Cambridge Press; 2005.

3. Spiegel MR. Mathematical Handbook of Formulas and Tables. McGraw-Hill; 1978.

4. Griffiths DJ. Introduction to Quantum Mechanics*.* Prentice-Hall; 1994.
